# Awareness, Knowledge, and Misperceptions Related to Nonalcoholic Fatty Liver Disease in a Community Sample of Mexican-Origin Women: A Mixed Methods Study

**DOI:** 10.3389/fpubh.2021.626428

**Published:** 2021-08-13

**Authors:** Kristin E. Morrill, Rebecca M. Crocker, Melanie D. Hingle, Cynthia A. Thomson, David O. Garcia

**Affiliations:** ^1^University of Arizona Cancer Center, University of Arizona, Tucson, AZ, United States; ^2^Center for Border Health Disparities, Health Sciences, University of Arizona, Tucson, AZ, United States; ^3^Department of Nutritional Sciences, College of Agriculture and Life Sciences, University of Arizona, Tucson, AZ, United States; ^4^Department of Health Promotion Sciences, Mel and Enid Zuckerman College of Public Health, University of Arizona, Tucson, AZ, United States

**Keywords:** Hispanic women, Mexican-origin, non-alcoholic fatty liver disease, NAFLD, liver disease, liver cirrhosis

## Abstract

**Introduction:** Mexican-origin women suffer disproportionate rates of nonalcoholic fatty liver disease (NAFLD) and research on how to tailor NAFLD treatment interventions for this population is lacking.

**Objectives:** The purpose of this study was to assess awareness, knowledge, perceptions, and information sources related to NAFLD in a community-based sample of Mexican-origin women.

**Methods:** This study employed a convergent parallel mixed-methods approach and consisted of a brief questionnaire (*n* = 194) and interviews (*n* = 26) among Mexican-origin women recruited from community-based settings including health fairs, churches, and community events. Participants were eligible if they identified as Mexican-origin, had a BMI ≥ 25 kg/m^2^, were 18–64 years of age, had the ability to speak, read, and write in English and/or Spanish, and provided informed consent. A purposeful sampling approach was used to recruit a subset of women (*n* = 26) with confirmed liver steatosis indicative of NAFLD (controlled attenuation parameter ≥280 dB/m) who completed the questionnaire. The twenty-six participants then completed one on one, in-depth semi-structured interviews to ascertain their knowledge and understanding of NAFLD.

**Results:** Qualitative findings revealed low awareness of risk factors for liver disease, NAFLD specifically. Knowledge of liver disease tended to center around cirrhosis, a condition many participants reported was prevalent in their families. Quantitative and qualitative findings both found information sources for NAFLD and liver disease to be predominantly friends, family, and media. Interviews revealed a misperception related to NAFLD risk that liver disease was only caused by high alcohol intake.

**Conclusion:** Low levels of NAFLD awareness and knowledge warrant the need for greater efforts to educate the general population, perhaps by integrating NAFLD education into existing type 2 diabetes educational campaigns and prevention interventions. Additionally, further elicitation research conducted in Mexican-origin adults is needed to elucidate key factors within behavioral-theory constructs that can be targeted in future interventions tailored to this unique population.

## Introduction

Over the past 30 years, global liver cirrhosis deaths have increased by over 50% (1). While major causes of liver cirrhosis vary by country (1), non-alcoholic fatty liver disease (NAFLD) has become an increasingly important risk factor for cirrhosis and has an estimated global prevalence of 25% (2). NAFLD, a metabolic disorder that refers to a spectrum of conditions ranging from benign non-alcoholic fatty liver through non-alcoholic steatohepatitis (NASH) to liver cirrhosis (3), is also the fastest growing indication for hepatocellular carcinoma (HCC), a highly fatal cancer (4, 5). Characterization of NAFLD includes the detection of hepatic steatosis by imaging or histology in the absence of other secondary causes of steatosis such as hepatitis C virus, significant alcohol consumption, use of certain medications, and related hereditary disorders (3).

In the United States, rates of NAFLD are consistently observed to be highest among Hispanic individuals, particularly Mexican Americans, where prevalence is 23% compared to 14% in non-Hispanic Whites (NHWs) and 13% in non-Hispanic Blacks (NHBs) (6). Importantly, these rates demonstrate greater prevalence in individuals with obesity and type 2 diabetes, conditions that also disproportionately burden Hispanics (7, 8). Similar disparities are observed in mortality from cirrhosis and liver cancer (5, 9). Since 2009, Hispanics have experienced among the greatest increase in deaths from cirrhosis (5). Additionally, incidence of HCC in Hispanics is double that in NHWs (4). Although incidence of liver cancer for men has stabilized over the past decade, new cases of liver cancer for women are rapidly increasing (10). Arizona, the site for this study, as well as Kansas, have experienced the greatest annual increase in liver cancer mortality since 1999 (5).

Mexican Americans remain the largest Hispanic subgroup in the United States comprising about 63% of the Hispanic population (11). Notably, chronic liver disease (CLD) and liver cirrhosis are the fourth leading cause of death in Mexico and the seventh leading cause of death in U.S. Hispanics (12, 13). However, differences exist in the most frequent cause of cirrhosis between the two countries. In Mexico, hepatitis C virus and alcoholic liver disease share as the leading causes of cirrhosis (13), while in the United States, NASH and alcoholic liver disease represent the most frequent causes (13). These differences can likely be attributed to a range of factors, including higher rates of obesity, type 2 diabetes, and abdominal obesity among US-based Mexican Americans, all risk factors for NASH (14).

Knowledge and awareness related to NAFLD remain low worldwide and in the United States (15, 16). Disease awareness and knowledge are integral components of health behavior theory— including the information- motivation-behavior (IMB) model (17), health belief model (18), and social cognitive theory (19)— that strives to elucidate why people do and do not engage in both positive and negative personal health practices. The IMB model was developed as an approach to understand the social and psychological factors that influence health-related behaviors represented as three separate, but correlated, constructs: information (facts and relevant heuristics), motivation (personal and social motivation), and behavior skills (objective ability and perceived self-efficacy). The IMB model asserts that information, motivation, and behavioral skills are key determinants and prerequisites for effective health-promoting action (17).

According to the IMB model, *information* serves as “an initial prerequisite” for successfully carrying out a given health behavior (17). However, information does not begin to motivate action until it is transformed into knowledge (information acquired by an individual) or awareness (generalized familiarities with a topic) (17). While efforts to distinguish between the concepts of awareness and knowledge within the public health literature have not always been clear or consistent, for the purposes of this article we employ the definition of awareness that situates it at the low end of a single knowledge continuum, representing general awareness that may later build to include more detailed and specific knowledge (20, 21). Level of knowledge has been shown to directly affect a wide range of mediating factors to effective behavior change including motivation, behavioral capability and objective skills, and perceived self-efficacy related to given health behaviors (17). Eliciting knowledge about specific health conditions can also highlight misperceptions that may subsequently shape health behaviors directly or indirectly through mediating factors such as perceived risk (22). Increasing awareness and knowledge of NAFLD risk is particularly critical given that the disease is both treatable in its initial stages and in many cases, preventable, with the adoption of health behaviors consistent with a healthy diet, weight management, and regular physical activity (3). Assessing NAFLD-related awareness, knowledge, and misperceptions in Mexican-origin women provides an opportunity to identify specific information assets and deficits that can be leveraged and targeted in future NAFLD education interventions, respectively (17).

There is a dearth of research on how to effectively tailor interventions for NAFLD prevention and treatment in U.S. Hispanic women. While previous studies have explored general risk factors for liver disease (23, 24) and hepatitis virus (23, 24), among Mexican-origin adults, no prior studies have focused specifically on NAFLD awareness, knowledge, and perceived health risks. This mixed methods study sought to fill in gaps by examining NAFLD-related knowledge and beliefs among high-risk Mexican-origin women within a community-based sample in Southern Arizona. The goal of this article is to present findings on awareness of NAFLD, sources of knowledge, and misperceptions about the disease in order to guide future NAFLD prevention and treatment intervention strategies specific to Mexican-origin adults in the United States.

## Materials and Methods

The study was conducted in Tucson, Arizona between May 2019 and March 2020 and employed a convergent parallel design. Quantitative and qualitative data were collected concurrently, analyzed independently, and then presented and interpreted in unison in the results and discussion sections. This mixed methods study design was used to identify areas of agreement (convergence), complementarity, or apparent contradictions (divergence) between the quantitative and qualitative data sets. A questionnaire was used to obtain the quantitative data while semi-structured interviews provided the qualitative data. Qualitative data were given higher priority than quantitative given the study's objective of gaining an in-depth understanding of knowledge, perceptions, and information sources related to NAFLD and liver disease. All study procedures were approved by the University of Arizona Institutional Review Board (IRB #1902380787).

### Quantitative

The purpose of the questionnaire was to assess awareness of fatty liver disease and conditions that can progress from it (i.e. cirrhosis), identify key liver disease information sources, and examine rates of familial diagnoses of conditions related to NAFLD (fatty liver, cirrhosis, and liver cancer).

#### Study Participation

Participants completed the questionnaire as part of an ongoing, cross-sectional study focused on liver disease. The main goal of this parent study was to estimate the prevalence of NAFLD and associated genetic, clinical, and behavioral risk factors among high-risk (Mexican-origin with overweight/obesity) adults residing in Southern Arizona. The study aimed to recruit 200 Mexican-origin men and 200 Mexican-origin women. As part of this study, participants attended a single study visit that took place in a clinic located in Tucson, Arizona that specialized in the treatment of liver disease. During the visit, participants were asked to complete a range of questionnaires including demographics, medical history, psychosocial factors, lifestyle behaviors, and awareness of NAFLD. Anthropometric measures including weight, height, and waist circumference were measured by bilingual, bicultural research assistants using protocol-driven methods and body mass index (BMI) was calculated by dividing weight in kg by height in meters squared (kg/m^2^). Additionally, all women completed a non-invasive transient elastography (FibroScan®, TE) to measure levels of liver steatosis or fat (decibels per meter or dB/m) and liver stiffness (kilopascals or kPa), a proxy for liver fibrosis, and had the opportunity, if interested, to discuss their results with one of two doctors, one of whom was Mexican-origin. Based on the results from the transient elastography, participants were categorized as having either less than mild, mild, moderate, or severe levels of hepatic steatosis. Related to liver stiffness, participants were categorized as having either no or little fibrosis, moderate fibrosis, severe fibrosis, or advanced fibrosis. At the end of the study visit, participants were compensated $25 for their time and were provided with a summary of their anthropometric assessment and Fibroscan® results as well as a list of local health care providers, including community-based health organizations, should they choose to seek medical care or review the results of their Fibroscan®. Participants were recruited using community-based approaches including face-to-face recruitment efforts at churches, community events and markets, and health fairs. During study recruitment, research staff shared with interested individuals the following NAFLD-related information: (1) NAFLD prevalence figures; (2) risk factors for NAFLD, including Mexican-origin Hispanic ethnicity; and (3) the reversible and preventable nature of the disease state. Recruitment occurred from May 2019 to March 2020 and study visits took place in an ongoing manner throughout this time period.

Eligibility criteria for the cross-sectional study are briefly described here: (1) self-identify as being of Mexican-origin; (2) 18-64 years of age; (3) BMI of ≥ 25 kg/m^2^; (4) ability to speak, read, and write in English and/or Spanish; and (5) ability to provide informed consent. Women were not eligible to participate if they (1) were currently diagnosed with uncontrolled vascular or metabolic disease (e.g., high blood pressure, type 2 diabetes); (2) consumed >14 standard alcoholic beverages/week; (3) were taking medication or dietary supplements known to affect body composition; (4) had any syndrome or disease known to affect body composition; (5) had participated in structured exercise, diet, or weight-loss program within six months of recruitment; (6) previously had bariatric surgery; (7) were pregnant or breastfeeding; (8) had previously been diagnosed with liver disease or liver cancer; (9) had a history of exposure to hepatotoxic drugs; and (10) had active, chronic gastrointestinal disorder (e.g. inflammatory bowel disease, ulcerative colitis, Chron's disease, celiac disease).

#### NAFLD Awareness and Related Conditions Questionnaire

A brief questionnaire was developed by K.M and D.G. to identify common sources of liver disease (fatty liver, cirrhosis, and liver cancer) awareness and knowledge. The following are the six questions included in the questionnaire: “*Has your doctor ever mentioned fatty liver? (Y/N)”, “Has one of your family members been diagnosed with fatty liver? (Y/N)”, “Have you ever heard of cirrhosis? (Y/N)”, “Has your doctor ever mentioned cirrhosis” (Y/N)*, “*Has one of your family members been diagnosed with cirrhosis? (Y/N)”, “Has one of your family members been diagnosed with liver cancer? (Y/N)”*. Participants completed the brief questionnaires between May 2019 and March 2020.

### Qualitative

#### Qualitative Research Design

Qualitative interviews were conducted in order to elicit awareness and knowledge related to NAFLD, identify common information sources, and identify perceptions related to NAFLD and NAFLD risk. Semi-structured interviews were chosen as the method for qualitative inquiry in order to examine individual knowledge of the disease and avoid bias from other participant responses and group interaction typical in focus group settings.

#### Study Participants

We recruited a subset of participants who completed the cross-sectional study who had also checked “yes” to a question asking if they were interested in participating in future semi-structured interviews. To be eligible for the qualitative interviews, participants needed to have liver steatosis scores of ≥280 dB/m at screening, indicative of NAFLD. We opted to recruit only those with suspected NAFLD to gather information specifically on those with the greatest risk of progressive liver disease. A purposeful sampling approach was used in an effort to recognize the effects of participant language preference and generation status on health knowledge and perception. Recruitment for the interviews took place from August 2019 to February 2020.

#### Sample Size

**Figure 1:**
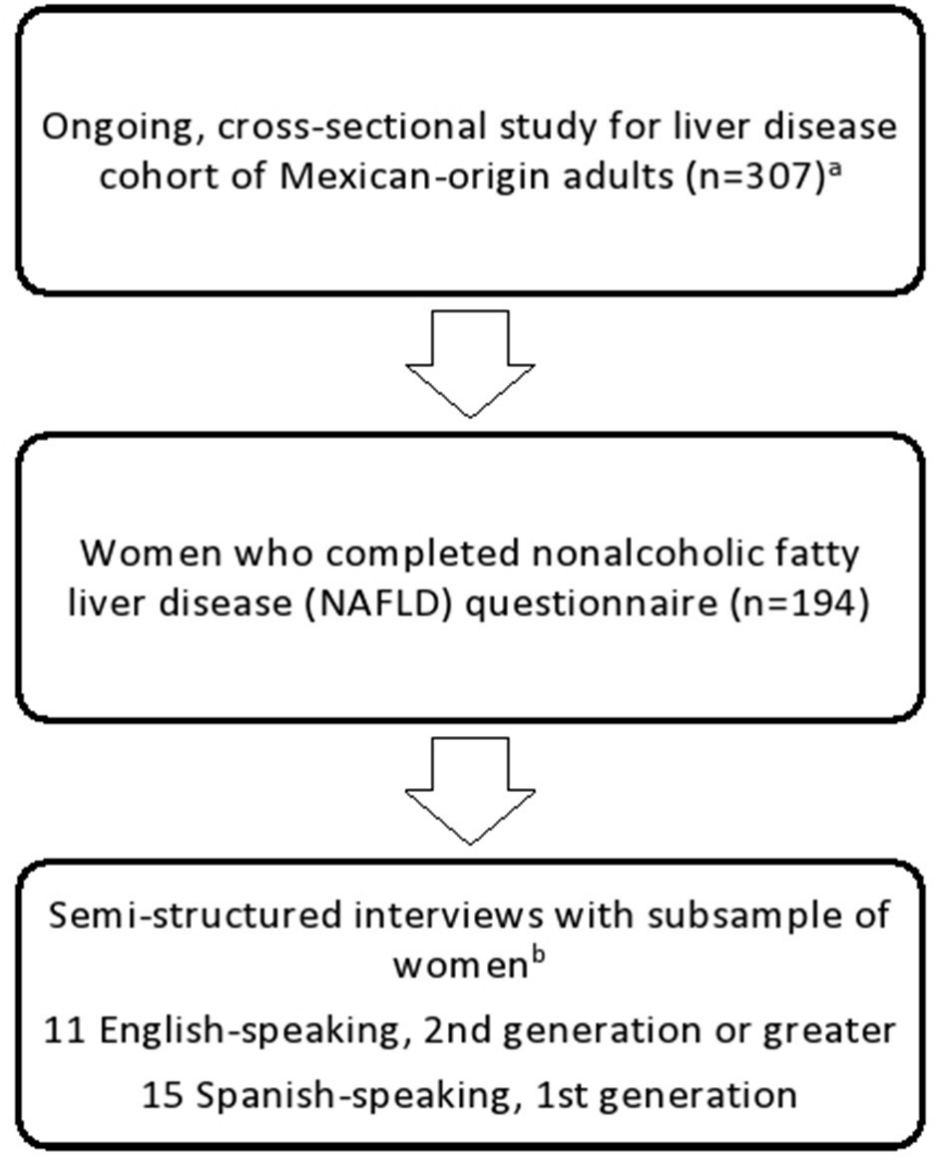
Recruitment flow diagram for study quantitative and qualitative methods. ^a^Eligibility criteria as follows: (1) self-identify as being of Mexican-origin; (2) 18–64 years of age; (3) BMI of ≥25 kg/m^2^; (4) ability to speak, read, and write in English and/or Spanish; (5) ability to provide informed consent. Women were not eligible to participate if (1) currently diagnosed with uncontrolled vascular or metabolic disease (e.g., high blood pressure, type 2 diabetes); (2) consumed ≥14 standard alcoholic beverages/week; (3) taking medication or dietary supplements known to affect body composition; (4) had any syndrome or disease known to affect body composition; (5) participated in structured exercise, diet, or weight-loss program within six months of recruitment; (6) previously had bariatric surgery; (7) pregnant or breastfeeding; (8) previously diagnosed with liver disease or liver cancer; (9) had a history of exposure to hepatotoxic drugs; and (10) had active, chronic gastrointestinal disorder (e.g., inflammatory bowel disease, ulcerative colitis, Chron's disease, celiac disease). ^b^Eligibility criteria as follows: liver steatosis scores of ≥280 dB/m at screening, indicative of NAFLD.

Out of the 194 women who completed the cross-sectional study, 91 women (91/194; 47%) were eligible and indicated they were interested in participating in the interviews. From these women, 45 (45/91; 49%) were contacted by study staff via telephone to schedule an interview. The final sample consisted of 11 US-born (as defined by 2^nd^ generation or above), English-speaking Mexican-origin women and 15 foreign-born (1^st^ generation), Spanish-speaking Mexican-origin women. A recruitment diagram for the quantitative and qualitative methods is presented in Figure 1. It has been proposed that a sample size of 20 people (25), but no more than 50 people (26), for interview-based qualitative studies is appropriate to reach data saturation at which point little new information is generated while accommodating the complexity of the data. In the current study, data saturation was achieved at 26 participants resulting in a final sample of 26 women.

#### Moderator Guide Development

The moderator guide used for the interviews was developed by a bilingual, bicultural doctoral student (K.M.) and two researchers with experience in qualitative research (M.H., and D.G.) and Hispanic population studies. Questions were developed taking into consideration the available literature on NAFLD awareness and knowledge. As part of the IMB model, it is advocated that *elicitation research*, in the form of open-ended questions, be conducted in a representative sample of the target population as the first-step to better understand and promote health behaviors through health interventions (17). Questions were specifically grounded within the “information” construct within the IMB model and were developed to elicit awareness and knowledge and identify specific information assets and deficits that could be targeted in future health interventions. Two University of Arizona (UA) Prevention Research Center Community Action Board (CAB) members who were Mexican-origin women contributed to the development of the moderator guide. The moderator guide was piloted with two members from the UA CAB and revised for clarity thereafter. Probes were included and used when needed. The English and Spanish moderator guides used are available as Supplementary Files 1, 2, respectively.

#### Semi-Structured Interviews

Bilingual and bicultural research staff led the in-person, semi-structured interviews; K.M. performed all English interviews and R.V. led all Spanish interviews. Interviews were conducted on weekdays and weekends to accommodate participant schedules. Interviewers followed the moderator guide. Interviews were designed to be, on average, between 45–60 minutes in duration. All interviews were audio-recorded. Summary sheets were drafted by the interviewer after each interview to refer back to during data analysis. Participants were compensated $25 for their time. All interviews took place at the University of Arizona Collaboratory for Metabolic Disease Prevention & Treatment in Tucson, AZ.

### Data Management and Analytic Methods

All participant demographic, clinical, and socioeconomic data were collected and managed using REDCap electronic data capture tools hosted at The University of Arizona (27, 28).

#### Quantitative

Descriptive statistics were used to summarize participant characteristics. Continuous variables were examined visually for normality and skewed variables (all except liver steatosis), were presented as median and interquartile range. Liver steatosis values were normally distributed and therefore presented as mean and standard deviation. All analyses were performed using R Studio version 3.6.2 (29).

#### Qualitative

Audio recordings were transcribed by bilingual, bicultural research assistants in the respective language of the participants. Spanish transcripts were then translated to English. Spanish phrases, idioms, and words that were contextually important were left in Spanish to avoid misrepresentation or loss of meaning. Transcripts were spot checked against recordings for accuracy by research assistants and then imported into NVivo 12 software which facilitated data management and analysis. Going into the coding process, only general topics that mirrored the topics to be covered in the semi-structured interview guide, such as “knowledge related to NAFLD”, “information sources”, and “perceptions related to NAFLD” were pre-identified and could be seen as overarching codes. While these topics provided a framework for the moderator guide and data analysis, our broad questions provided space for themes and codes to emerge emically from the participants' own words. Codes generated from the data itself shaped the conclusions drawn from the interviews.

Coding was conducted by a trained doctoral student (K.M) and a qualitative researcher with extensive coding experience (R.C). To analyze the data, K.M. and R.C. employed Braun and Clarke's six phases of thematic analysis. First, transcripts were reviewed to become familiar with the data. Next, K.M. and R.C. independently coded three transcripts and met to discuss any discrepancies until the initial set of codes were developed. A codebook was drafted and continuously updated as codes were revised or added. Coding was an iterative process and transcripts were reread to test the applicability of the coding scheme. A series of conversations took place between K.M and R.C. where codes were discussed and combined into candidate themes. Themes were then reviewed to ensure they adequately reflected the data and codes.

## Results

Demographic and clinical characteristics of the 194 Mexican-origin women who completed the questionnaire are summarized in Table 1. Participants ranged in age from 19 to 64 years with a median age of 47 years. The majority of women (70%) were 1^st^ generation and primarily spoke Spanish at home (73%). Most women were married or living with a domestic partner (69%) and over half of the women (61%) were employed. Participants' level of formal education fell into five categories: less than high school (19%), high school or GED (32.5%), some college (14.4%), Bachelor's degree (8.8%), and graduate degree or higher (25.2). Fifty-eight percent of women had health insurance and 64% reported currently having a primary care provider. The median BMI in our sample was 32.3 kg/m^2^, which fell within the obese range. Liver steatosis in our sample ranged from 164 dB/m to 400 dB/m, corresponding to a range of low steatosis to severe steatosis. Liver stiffness ranged from 2.2 to 38.3, corresponding to a range of no or mild liver scarring to advanced liver scarring. Results from the questionnaire are summarized in Table 2 and a joint display of quantitative and qualitative findings can be found in Table 3.

**Table 1:** Characteristics of Mexican-origin female participants (*n* = 194).

	**Total Sample (** ***n*** **=** **194)**	
**Characteristics**	***n* (%)**	**Median (IQR)**
Age (yrs)		47.0 (38.0, 54.0)
Weight (kg)		82.9 (74.0, 93.1)
BMI (kg/m^2^)		32.3 (29.5, 35.8)
Liver steatosis (CAP)^a^ Less than mild Mild Moderate Severe	28 (14.5) 35 (18.0) 34 (17.5) 97 (50.0)	288.0 (48.6)
Liver stiffness (kPa)^b^ No or mild scarring Moderate Severe Advanced	163 (84.0) 25 (12.9) 4 (2.1) 2 (1.0)	5.0 (4.3, 6.1)
Currently married or living with domestic partner, *n* (% yes)	133 (68.6)	
Employed, *n* (% yes)	118 (60.8)	
Health insurance, *n* (% yes)	112 (57.7)	
Primary care provider, *n* (% yes)	118 (60.8)	
Self-reported diabetes, *n* (% yes)	21 (10.8)	
Primary language spoken at home		
English	52 (26.8)	
Spanish	142 (73.2)	
Foreign born	136 (70.1)	
U.S. born	58 (29.9)	
Generation status^c^		
1^st^ generation	136 (70.1)	
2^nd^ generation	35 (18.0)	
3^rd^ generation	7 (3.6)	
4^th^ generation or greater	16 (8.3)	
Income		
< $29,999	100 (51.5)	
$30,000–59,000	68 (35.1)	
>$60,000	26 (13.4)	
Last grade completed		
Less than high school	37 (19.1)	
High school or GED	63 (32.5)	
Some college	28 (14.4)	
Bachelor's degree	17 (8.8)	
Graduate degree or higher	49 (25.2)	

a *Liver steatosis expressed as mean [standard deviation (SD)] given normal distribution. Possible CAP values for liver steatosis ranged from 100 to 400. Steatosis grade was categorized as the following: less than 238 = less than mild steatosis, 238–260 dB/m = mild steatosis, 261–290 dB/m = moderate steatosis, higher than 290 dB/m = severe steatosis*.

b *Liver stiffness used to estimate levels of liver fibrosis. Possible values ranged from 2 kPa to 75 kPa. Fibrosis score was categorized as the following: 2–7 kPa = no liver scarring or mild liver scarring, 7.1–10 kPa = moderate liver scarring, 10.1–14 kpa = severe liver scarring, 14 or higher = advanced liver scarring*.

c *Generation status categories were described as the following: 1^st^ generation = born in Mexico, 2^nd^ generation = born in United States, either parent born in Mexico, 3^rd^ generation = born in United States, both parents born in United States, all grandparents born in Mexico, 4^th^ generation = born in United Status, both parents born in United States, at least one grandparent born in Mexico with remainder born in the United States*.

**Table 2:** Participant responses to nonalcoholic fatty liver disease awareness and related conditions questionnaire (*n* = 194).

**Question**	***n***	**%**
Has your doctor ever mentioned fatty liver?		
Yes	34	17.5
No	160	82.5
Has one of your family members been diagnosed with fatty liver?		
Yes	44	22.7
No	150	77.3
Have you ever heard of cirrhosis?		
Yes	165	85.1
No	29	14.9
Has your doctor ever mentioned cirrhosis?		
Yes	14	7.2
No	180	92.8
Has one of your family members been diagnosed with cirrhosis?		
Yes	35	18.0
No	159	82.0
Has one of your family members ever been diagnosed with liver cancer?		
Yes	25	12.9
No	169	87.1

**Table 3:** Joint display of quantitative and qualitative findings.

**Domain**	**Survey results**	**Qualitative illustrative quotes**	**Interpretation of mixed methods findings**
NAFLD Awareness and Knowledge	22.7% of participants had a family member diagnosed with fatty liver 18% of participants had a family member diagnosed with cirrhosis 12.9% of participants had a family member diagnosed with liver cancer	“*I actually wasn't aware of this disease. Before getting the study done, I didn't know about it.”* “*I am going to be honest, beforehand, I had heard something about fatty liver, but I don't remember the specifics.”* “*I didn't know anything about [NAFLD] prior to getting the study done, this was when I started going online and reading more about it.”*	Low NAFLD awareness and knowledge were observed despite high rates of familial diagnoses. Among participants, NAFLD awareness did not generally translate to NAFLD knowledge.
Information Sources for Liver Disease	82.5% of participants had never heard of fatty liver from their doctors 92.8% of participants had never heard of cirrhosis from their doctors	“*I have heard about [fatty liver] and I think I got it from watching movies on YouTube.”* “*Um, just doing some reading online because I would always pop up on my…my um…e-mails sometimes. And my father died from liver disease. I didn't know him, but they say he wasn't an alcoholic. So that got me kind of thinking.”* “*So my brother has it and I had never heard of it before that. And so I was like googling and I just…I just didn't really know too much other than just it's genetic.”* “*And it's something that you don't learn about. Like I have a four-year degree and a lot of it was like I had to take nutrition classes. I had to take all these science classes. I had to take a lot of bio classes. Nobody ever told me this.”*	NAFLD information sources were predominantly informal including family, friends, and media sources. Some participants with a family history of liver disease conducted their own research via the internet, television, and/or reading to learn more about the disease.
Misperceptions Related to Liver Disease	Note: Because misperceptions related to liver disease risk factors emerged emically during the qualitative phase (participants completed the interviews after their study visit), there were no related questions included in the survey.	“*I was under the impression that people who have liver problems are the ones who drink alcohol.”* “*I was always under the impression that issues that had to do with the liver was due to drinking alcohol and I've also heard of liver cirrhosis and I thought it was because a person was drinking excessively.”*	Results of the qualitative interviews indicated that the belief that only those who consume alcohol were at risk of the broader spectrum of liver disease seemed to contribute to lower levels of perceived risk among women.

### Fatty Liver and NAFLD Awareness and Knowledge

One of the primary goals of the interview was to assess NAFLD awareness among women and identify knowledge related to the condition. Specifically, women were asked the question, “*Can you tell me a bit about what you know about non-alcoholic fatty liver disease (NAFLD)?”* When presenting quotes below, we have chosen to include the following participant information: Spanish/English (language preference) and age. A summary of themes, codes, and illustrative quotes from the qualitative interviews can be found in Supplementary File 3.

Although results from the questionnaire indicated that almost one quarter of the sample had family members who had been diagnosed with fatty liver disease, overall knowledge about NAFLD was very low among those who participated in the interviews. For almost half of the women, initial conversations that took place during recruitment with study staff were the first time they had heard about NAFLD or fatty liver more broadly. A 26-year old, Spanish-speaking woman described meeting with study staff during a back-to-school community event, “*I wasn't really aware of this topic until the day they were giving out backpacks. I learned information about this study there because I didn't know anything about it beforehand. I also knew that the liver sometimes fails, but I never really knew why.”(S, 26)* A 56-year old, Spanish-speaking participant shared a similar experience, saying: “*I actually wasn't aware of this disease. Before getting the study done, I didn't know about it.” (S, 56)*. Additionally, while a few women stated they had heard about fatty liver before, they did not recall any detailed information related to the condition. One 61-year old Spanish-speaking woman with a family history of cirrhosis said, “*I am going to be honest, beforehand, I had heard something about fatty liver, but I don't remember the specifics.” (S, 61)*

English-speaking participants more frequently stated they had heard of NAFLD before the study as compared to their Spanish-speaking counterparts. Spanish-speaking participants more frequently made statements like “*No, I didn't even know it existed” (S, 33)*; “*To be honest with you, I didn't know this disease existed” (S, 55); “I didn't know anything about it prior to getting the study done” (S, 50);* and “*I actually wasn't aware of this disease…” (S, 56)*. In contrast, among English-speaking women, statements about pre-study NAFLD awareness included “*I just know I've heard it mentioned” (E, 53); “I know it existed but…” (E, 38)* and “*I've read it on my own searching” (E, 42)*. However, despite greater awareness of NAFLD among English-speaking women, knowledge related to risk factors, etiology, symptoms, and general understanding of the disease was very limited among all women.

Pre-study knowledge about liver disease mostly centered around cirrhosis, not NAFLD. As one 50-year old, English-speaking participant summarized, “*Um…before this, I really didn't know anything about it [NAFLD]…I had only heard of…like cirrhosis of the liver. That's it. I had never heard of fatty liver.” (E, 50)* However, results from the questionnaire indicated that only a small percentage of women (7%) had heard of cirrhosis previously from their doctors. Among the women who had heard of fatty liver before the study, most were not aware of the association with dietary intake and other lifestyle behaviors and had limited knowledge of specific details related to the disease.

A few participants contrasted the lack of general awareness of NAFLD to the comparatively higher level of awareness of other chronic diseases, such as diabetes, heart disease, and cancer. In doing so, a few women explained this lack of awareness as stemming from the liver not being a common area of concern for most participants and their families. One 26-year old Spanish-speaking participant shared that she had first heard about NAFLD at a health fair where she engaged with study staff who were recruiting for the study on-site. During her interview, she stated: “*This is something one isn't aware of because one doesn't normally pay attention to the liver. Everyone says ‘oh, I have diabetes because it runs in the family', but no one pays attention to the liver.” (S, 26)*. Another participant shared, “*You know [about] like heart disease, diabetes, and all that before your liver, there's not a lot of things known about it.” (E, 38)*.

While not a common theme, three participants specifically mentioned the link between medication use and liver disease. In two of these cases, the women mentioned that medication had contributed to the progression of liver disease in a now-deceased family member, although details related to the medication and the family member's experience were not known. When recalling the loss of her brother, one participant shared, “*To be honest with you, I didn't even know this disease existed. One of my brothers passed away three years ago from fatty liver disease due to a specific medication he was taking but I'm not quite sure. I'm not sure which types of medications are the ones that harmed his health.” (S, 55)*.

### NAFLD Information Sources

A second central aim of the qualitative research was to explore where participants were obtaining information related to fatty liver and liver health more broadly. After eliciting knowledge of NAFLD, interviewers asked the question, “*How did you learn about this?”*

Most participants who had heard of NAFLD before the study had been exposed to it through family and friends. This finding was supported by the high rates of familial diagnoses of liver disease observed from the results of the questionnaire. Specifically, 23% (44/194) of participants had family members who had been diagnosed with fatty liver, 18% (35/194) with cirrhosis, and 13% (25/194) with liver cancer. When asked to share what she knew about NAFLD, one 23-year old, English-speaking participant with a family history of fatty liver disease and liver cancer shared, “*So my brother has it and I had never heard of it before that.” (E, 23)*. Another young English-speaking participant was visibly distressed when she shared:

*Well, um I mean…Personally, I just recently um lost a family member to liver cancer and um I mean I know in the past he…I mean…let's just say he was a drunk. He would drink like…a lot. So I just heard that you know that affects it. So that's one of the…how I know a little bit like oh well maybe his drinking problem could have caused this cancer now, like developed it, or affected his liver. (E, 27)*.

While family history of liver disease afforded participants basic awareness of different conditions affecting the liver, in most cases participants' knowledge and understanding of the diseases were minimal. Participants who had family members with liver disease recalled very limited information related to the exact cause or nature of their family member's condition. When asked, one English-speaking participant shared how she first heard about NAFLD when she stated, “*Mostly from talking with you because I have no idea. My husband said he had fatty liver disease, but he didn't think it was a big thing. And then my daughter was diagnosed with it years ago and she's in her 20s.”(E, 62)* Likewise, another woman expressed confusion about the cause of a recent family member's passing, saying:

*A week ago, my husband's first cousin who was the only daughter passed away from a liver disease. But she had some other issues with her bones and joints, and I didn't really understand exactly what happened. I called my mother-in-law and she told me that the medications she was taking for her bones and joints affected her liver. Therefore, I don't remember what else happened, but I do know she passed away due to her liver. (S, 50)*.

Notably, both our quantitative and qualitative findings revealed that only a minority of study participants learned about NAFLD from formal sources such as medical staff, formal education, or health campaigns. Only one participant mentioned having spoken to her doctor about NAFLD during the interviews, and over 80% of participants who filled out the questionnaire checked never having heard of fatty liver from their doctors. Participants also noted a lack of information about NAFLD from formal health informational campaigns. One 38-year old English-speaking participant mentioned, “*I know it existed but I never like see it on TV or anything like that like in a big way like ‘oh my god you have to take care of it.” (E, 38)* Even participants who had completed higher education noted a dearth of information related to liver disease. A 26-year old English-speaking participant who had completed both college and graduate school expressed frustration that she had never learned of NAFLD at school. She shared, “*And it's something that you don't learn about. Like I have a four-year degree and a lot of it was like I had to take nutrition classes. I had to take all these science classes. I had to take a lot of bio classes. Nobody ever told me this.” (E, 26)*.

Many participants described how they had conducted their own research via the internet, television, or reading in order to learn more, particularly those with a family history of liver disease. One 42-year old, English-speaking participant shared “*I've read it on my own searching just because my family has a history of liver disease. Both my parents passed away because of liver. My dad had liver cancer and then my mother had liver cirrhosis…Because of their history and what went on with them, which was recent, it made me want to look into it, so I started reading up on it.” (E, 42)*. In some situations, participants' exposure to NAFLD seemed fleeting, particularly when the person had read about it in an email or in a brief appearance on television or a video. It was common for participants to express uncertainty about where they had heard the information or recall specifics of what they had learned. One woman shared, “*I have heard about it and I think I got it from watching movies on YouTube.” (S, 37)* For some women, learning about NAFLD through their participation in the study had inspired them to perform their own research to learn more about the disease. As one 50-year old, Spanish-speaking participant summarized: “*I didn't know anything about it prior to getting the study done, this was when I started going online and reading more about it.” (S, 50)*.

### Perceptions Related to NAFLD and Relation to Perceived Risk

During the course of the interviews, one key misperception related to NAFLD emerged that led women to misinterpret their risk for liver disease. To explore NAFLD perceptions, we asked the women two consecutive questions: “*Is there anything you would like to share that you learned from your participation in this research study?”* and “*Was there something you learned from the study that was different from what you had previously thought?”*

During the interviews, several participants expressed how they believed that only people who consumed alcohol were at risk for liver disease. For example, one young English-speaking woman stated that through her participation in the study, “*I learned that you could have a fatty liver without alcohol. That's, I honestly like that was one of my biggest I guess breakthroughs.” (E, 26)*. Participants generally connected alcohol consumption with cirrhosis of the liver, a condition that was recognized as a serious health threat. When asked to share what she knew about NAFLD before the study, one 43-year old Spanish-speaking participant stated, “*Well, I didn't know this disease existed, specifically on this topic. I was under the impression that people who have liver problems are the ones who drink alcohol. This usually leads to cirrhosis, something of that sort. But I hadn't heard about fatty liver.”(S, 43)*.

The misperception that only those who consume alcohol were at risk of liver disease contributed to some women expressing low levels of perceived risk for the broader spectrum of liver diseases. Given that many women in the sample reported low levels of alcohol consumption, they did not consider themselves at-risk for liver disease. As such, during the course of the study, many women expressed they felt surprised when they learned that other dietary factors besides alcohol could contribute to the development of liver disease. During her study visit, one 62-year old, English-speaking participant described her feelings after learning she had severe liver steatosis and moderate liver scarring: “*I was surprised considering that I don't drink and that I don't have any of those external…that would affect liver. I mean either drug use or whatever that would affect. But I don't have those in my life.”(E, 62)*. Another participant recalled her response to seeing her liver ultrasound results, which indicated a high level of liver steatosis, “*So I was thinking like well, I don't drink, I've never drank in my life. So it's like, well, it could also be a part of genetics or it could be like the way I eat. So, I know it wasn't triggering because of the alcohol because I don't consume it.”(E, 27)*.

## Discussion

Major themes that emerged from the data were the following: (1) liver disease knowledge centered around cirrhosis, a condition that was reported by participants to be prevalent in their families; (2) while informal information sources such as family history of liver disease afforded some participants basic awareness of different conditions affecting the liver, in most cases, this awareness did not translate to knowledge and understanding of the diseases, including NAFLD; and (3) the common misperception that only those who consume alcohol were at risk of liver disease which seemed to contribute to low perceived risk for the broader spectrum of liver diseases given many of the women reported not consuming alcohol.

Findings from the interviews revealed low pre-study NAFLD awareness and knowledge among women. Approximately half of the participants described learning of NAFLD and fatty liver disease for the first time during study recruitment into the parent study. Information sources for knowledge related to liver health were predominantly informal, derived from conversations with family and friends or brief exposures via the television and internet. This finding was consistent with our quantitative data which showed high rates of liver disease diagnoses, including fatty liver, cirrhosis, and liver cancer, among participants families and very low rates of participants' having heard of NAFLD through a medical practitioner. As such, misperceptions about NAFLD were common among study participants, most notably the belief that only those who consumed alcohol could develop liver disease.

While our findings support prior data showing overall low rates of NAFLD awareness among the general population in the United States (15, 16) and around the world (30, 31), to our knowledge this is the first study to document this phenomenon specifically among US-based Mexican-origin adults. Study participants displayed some knowledge related to liver disease (specifically cirrhosis). However, they shared very little prior knowledge of the nature and risk factors of NAFLD specifically. This finding is particularly important given the high rates of NAFLD in Mexican-origin adults relative to other Hispanic and non-Hispanic subgroups in the United States (6, 32). A lack of awareness of the breadth of risk factors for liver disease, particularly non-alcoholic risk factors, has been previously reported in US-based and Mexican adults (23). Several study participants noted that knowledge of NAFLD and liver disease more generally paled in comparison to community-level awareness of type 2 diabetes and cancer, which are generally well-known.

Of particular note, our qualitative and quantitative results indicate a dearth of NAFLD and cirrhosis-related information dissemination from medical professionals to this high-risk population. This finding may reflect the well-documented barriers to health care access among US-based Hispanics and particularly foreign-born Hispanics, an issue that produces and compounds grave health disparities (33), or a lack of specific discussion by health care providers of this health risk when medical interactions do occur. Moreover, over 70% of our participants were 1^st^ generation and health care access in Mexico has also been historically limited for a wide swath of Mexican citizens, particularly those born in low-income and rural communities and those who grew up prior the advent of universal health care in Mexico in 2004 (34). These binational barriers to health care access likely contribute to the comparatively low NAFLD awareness among Mexican-born populations as compared to their US-born counterparts (23), a finding supported by this study. Moreover, the lack of information dissemination from doctors to study participants may reflect the incomplete NAFLD literacy among health professionals in Mexico (24) and the United States (35, 36), which can impede effective communication with patients about this critical disease state.

In addition, the results of this cross-sectional, mixed methods study reflect the potential need for alternative strategies to communicate NAFLD risk in Mexican-origin women. Informal networks for health information dissemination are common and may prove effective in increasing knowledge of NAFLD health risks among Mexican-origin populations in the United States. The importance of family, friends, and mass media as sources for general health, diabetes, and cancer information in the Hispanic community has been well documented (37, 38). However, given the low general awareness about NAFLD and the lack of formal information sources for liver disease information targeting Hispanics, in concert with NAFLD's immense and disproportionate burden among Mexican-origin populations, testing of evidence-based strategies for information dissemination in regards to this disease state may be particularly relevant. While most participants cited family as their primary source of information about liver disease, even in cases of NAFLD diagnosis within the family, these familial information sources generally did not correlate to high degrees of specific knowledge about the disease.

The overall low NAFLD awareness and knowledge observed in this study population carry important implications for how we understand individuals' assessment of risk and vulnerability for NAFLD in Mexican-origin populations. Risk perception has been found to be a critical component of several theories for health behavior change given its association with engagement in health behaviors (22). Although we did not directly assess perceived risk for NAFLD or other liver diseases in our study, we observed that the lack of information about NAFLD produced misperceptions that affected the women's perceived risk of liver disease. Perception of risk for a disease has been shown to be higher when participant family members have been diagnosed with the disease (39). The misperceptions related to non-alcoholic risk factors for NAFLD may have stymied the increase of perceived risk that may have come from the high rates of familial diagnoses of fatty liver, cirrhosis, and liver cancer among participants. Further efforts to robustly characterize risk perceptions related to NAFLD, cirrhosis, and HCC among Mexican-origin women are needed.

Specifically, this study adds to our understanding of how high levels of alcohol-induced cirrhosis in Mexican-origin populations impact liver health knowledge and perceptions among US- based Hispanics. The majority of study participants made a clear association between liver disease and alcohol and were far more likely to have heard of alcohol-related liver disease than NAFLD. Similarly, high community-level knowledge of excessive alcoholic consumption as a risk factor for liver disease among US-based and Mexican adults was reported by Flores et al. (23). This finding may be attributed, in part, to the fact that Mexico ranks first in the world for alcohol-induced liver cirrhosis and death (40). Study participants cited high rates of cirrhosis in their families and recognized the gravity of liver disease.

The disproportionate burden of liver-related mortality among Mexican-origin people is not due to alcohol alone (13, 14). It is possible the historic prevalence of alcohol-induced cirrhosis in Mexico acted to obfuscate other important risk factors for liver disease, producing misperceptions regarding the risk factors and causal mechanisms of liver disease. A number of participants stated that, prior to study participation, they had believed that only those who consume alcohol were at risk of developing liver disease and had very limited knowledge of non-alcoholic lifestyle risk factors for liver disease. This belief contributed to overall low pre-study perceived risk among our sample of women. Our findings were consistent with those of Alemany-Pagès et al. (30), where Portuguese participants with type 2 diabetes displayed similar high cirrhosis awareness but had poor understanding of its connection to fatty liver (30).

### Implications of Research

The relatively low levels of health care access and utilization in US-based Hispanics compared to other racial subgroups is relevant when considering the clinical implications of this study (41). This reduced access limits opportunities for information dissemination and makes it critical that practitioners use patient interactions as a means to address potential misperceptions related to risk for NAFLD, particularly the role of non-alcoholic etiological factors in the development of liver diseases. In addition, health care providers should harness the high level of community awareness of type 2 diabetes among Hispanic immigrants (42) in order to inform and educate their patients about NAFLD risk as well as the shared risk factors between the two disease states (43, 44).

Moreover, the results of our study offer important lessons for how best to structure public health interventions. Community based intervention strategies for NAFLD prevention and treatment may be particularly useful rather than relying on clinic-based settings alone, given low rates of health care access and utilization in this community (33). However, in studies that do take place in clinic-based settings, NAFLD education can be integrated into existing programs for diabetes prevention given the shared risk factors related to obesity and diet. Additionally, interventions should be geared toward the family as a whole rather than the individual given that families are often conduits for health knowledge and that NAFLD risk is patterned along lines of genetic susceptibility (45, 46). Indeed, even after controlling for important confounders, one study found that first degree relatives of patients with NAFLD-cirrhosis had 12 times greater risk of advanced fibrosis (46). These data suggest that targeting the family as a unit both for health interventions and data collection related to family history of liver disease may prove fruitful.

Finally, low levels of NAFLD awareness and knowledge necessitate efforts to raise awareness of the spectrum of liver diseases and their associated risk factors. Targeting health knowledge through campaigns (47) and health education interventions (48–50) have been shown to significantly increase engagement in health-promoting behaviors among Hispanics in the United States. This approach was previously used to raise awareness of prediabetes and has been remarkably successful based on those reported to have taken the test and went on to locate a lifestyle change program (51). A feasible approach to disseminate NAFLD health information may be to incorporate NAFLD education into existing type 2 diabetes campaigns.

### Future Research

Future qualitative research is necessary to add nuance to our understanding of the potential impact of NAFLD in this high-risk population and how best to address it. Areas of additional research should include the exploration of intrafamilial communication specifically focused on liver disease to expand our understanding of the way in which health information is shared between family members and the effects this communication channel has on disease perception. Additionally, future research should more thoroughly evaluate NAFLD health risk in the “motivation” and “behavior” constructs of the IMB model (17). An idea would be to use *a priori-*designed survey items to explore associations between perceived and objective risk of liver diseases in Mexican-origin adults and how these factors affect motivation to adopt dietary behaviors found to protect against NAFLD. Finally, additional research into awareness and knowledge of NAFLD in Mexico would greatly inform our understanding of the binational transmission of information related to this disease state. Taken in concert, these findings would be useful in guiding the development of future culturally-sensitive lifestyle interventions aimed at prevention, early diagnosis, and treatment of NAFLD in this at-risk population.

### Strengths and Limitations

A key strength was the grounding of our research questions within the IMB model. Specifically, we identified key information deficits and misperceptions related to NAFLD health risk. Another strength was the decision to focus on a more homogeneous group of women with some shared life experience in relation to residing in Southern Arizona and being female and classified with overweight/obesity. Additionally, the use of a mixed-methods approach allowed the researchers to provide a voice to the experiences of study participants that served to contextualize the results of the quantitative questionnaire. In an effort to acknowledge the well-documented associations between acculturation and health perceptions in US Hispanics (52), we also explored potential differences in responses using acculturation proxies.

This study also has several limitations. First, the presentation of NAFLD-related prevalence and risk factors during study recruitment biased survey answers related to pre-study NAFLD knowledge and information sources. This led the researchers to exclude responses to related survey questions which would have provided more expansive data regarding generalized knowledge and awareness of NAFLD in the study population. Additionally, the questionnaire used in the study was not developed in a way to assess a singular construct, which limited our ability to evaluate the reliability of the questionnaire. Instead, questions were included to triangulate findings related to awareness and information sources from the qualitative interviews. It is also important to consider the potential for recall bias to have influenced participants' responses, particularly with questions that asked participants to recall previous familial diagnoses and sources of information. Lastly, we must acknowledge the limitations of participants' reports of familial diagnoses of liver disease which we did not confirm by medical records or other medical source.

To summarize, both quantitative and qualitative findings revealed low awareness of NAFLD among Mexican-origin women recruited from a Southern Arizona community. Participants were most likely hear about liver disease through their family and friends, often due to a family member's medical diagnosis. A lack of formal information sources specific to NAFLD may have contributed to the development of misperceptions related to common risk factors for liver disease, which in turn contributed to a lower perceived risk among the women. Findings suggested that greater efforts should be placed on raising awareness of NAFLD and non-alcoholic risk factors for liver disease more broadly, particularly among populations at high risk for progressive non-alcoholic liver disease and low access to health care. These findings will inform upon the development of future interventions seeking to prevent or treat NAFLD in Mexican-origin adults.

## Data Availability Statement

The original contributions presented in the study are included in the article/Supplementary Material, further inquiries can be directed to the corresponding author/s.

## Ethics Statement

The studies involving human participants were reviewed and approved by The University of Arizona Institutional Review Board (IRB #1902380787). The participants provided their written informed consent to participate in this study.

## Author Contributions

KM and DG devised the study. KM, MH, CT, and DG developed the moderator guides. KM and RC coded and analyzed all semi-structured interviews and wrote the final manuscript. DG supervised the project. All authors provided feedback to each manuscript draft.

## Conflict of Interest

The authors declare that the research was conducted in the absence of any commercial or financial relationships that could be construed as a potential conflict of interest.

## Publisher's Note

All claims expressed in this article are solely those of the authors and do not necessarily represent those of their affiliated organizations, or those of the publisher, the editors and the reviewers. Any product that may be evaluated in this article, or claim that may be made by its manufacturer, is not guaranteed or endorsed by the publisher.
