# Supplementary material for: Awareness, Knowledge, and Misperceptions Related to Nonalcoholic Fatty Liver Disease in a Community Sample of Mexican-Origin Women: A Mixed Methods Study
Source: Front Public Health. 2021 Aug 13;9:626428. doi: 10.3389/fpubh.2021.626428 (PMC8415027; doi:10.3389/fpubh.2021.626428)
Supplement: Supplementary file 1 [file Data_Sheet_1.pdf]

Supplementary Material

Supplementary File 1: English Semi-Structured Interview Moderator Guide

Introductions

Hello, my name is [first name]. Thank you for being here with me today. I have us scheduled for 60-90 minutes together. Does that still work for you? I want to make sure we honor our time constraints today. Therefore, while I encourage you to elaborate on your answers to my questions, there may be times when I redirect, so that we are able to cover all of the topics while we are together. At the end of this interview, you will be compensated $25 for your time.

To start, I would like to remind you that with this study we are hoping to learn more about what the community knows about nonalcoholic fatty liver disease as well as perceptions and beliefs surrounding this condition. We are also interested in learning more about meal patterns and perspectives of healthy eating. The next set of questions will explore your thoughts and beliefs surrounding the idea of genetic testing. With the last set of questions, we are interested in gathering suggestions from community members on how to develop programs to treat nonalcoholic fatty liver disease and, specifically, how we can tailor these programs to meet the needs of Mexican-origin women.

Your participation in this interview is voluntary and you may stop at any time you wish.

When we start the interview, I would like to begin voice recording our conversation. Do I have your permission to do this? I will also be taking some notes as we go, but the tape recording will ensure that I have a record to refer to about our conversation. Your thoughts and experiences are really important to us, so we want to do our best to capture your words accurately. I would like to remind you that everything that you say will be confidential. All recordings will be securely stored here in our research office (The Collaboratory for Metabolic Disease Prevention and Treatment). The research team will summarize this interview and use your contribution to develop results and conclusions for the research, but any information that can identify you will be removed.

I will now begin recording. [Begin recording]

Part 1: Assessing information/knowledge, motivation, and behavioral skills related to NAFLD health risk

Knowledge, Perceptions, Beliefs Related to NAFLD and NAFLD health risk

"So for this first section, I'm going to ask you some questions related to your knowledge and beliefs surrounding nonalcoholic fatty liver disease (NAFLD)."

1. (Knowledge/Beliefs) "Can you tell me a bit about what you know about nonalcoholic fatty liver disease (NAFLD)?"
    a. (Information sources) "How did you learn about this?"
2. (Perceived Susceptibility) "Do you believe that there are certain people who are more likely to get nonalcoholic fatty liver disease? OR Can you tell me about what might make someone more likely to "get" nonalcoholic fatty liver disease?"
3. "Is there anything you'd like to share that you learned from your participation in this research study?"
    a. "Was there anything you learned that was different from something you previously thought?"
    -

Part 2: Experience Receiving Transient Elastography (Fibroscan)

"Thank you for sharing. For this next section, I'd like to now bring you back to the day of your study visit. Specifically, I want you to think about your experience receiving the liver ultrasound."

1. "What were some of your initial thoughts upon seeing your liver ultrasound results and speaking with the provider (doctor)?"
    a. "Was the information from the provider useful?"
    b. "Are you interested in receiving another free ultrasound in the future?"
2. "Who did you share results with, if anyone?"
3. "Can you tell me whether you took any specific action or actions based on the ultrasound or as a result of your discussion with the provider (doctor)?"
4. "If there had been an opportunity to learn more about your risk of nonalcoholic fatty liver disease by having your blood drawn or providing a urine sample, would you have been interested? Why or why not?"

Closing Statements

Supplementary File 2: Spanish Semi-Structured Interview Moderator Guide

Introducción

Hola, mi nombre es [nombre]. Gracias por estar aquí hoy. Tenemos programados aproximadamente 60 a 90 minutos para la entrevista de hoy. ¿Eso todavía funciona para usted? Quiero asegurarme de que respetamos nuestro tiempo asignado. Por lo tanto, aunque le pedimos que elabore sus respuestas a mis preguntas, puede haber ocasiones en las que la redirija hacia el tema principal para que podamos cubrir todos los temas que tenemos planeados para el día de hoy. Al final de esta entrevista, se le compensará $25.00 por su tiempo.

Para comenzar, me gustaría reiterar que con este estudio esperamos aprender más sobre lo que la comunidad sabe sobre la enfermedad del hígado graso no alcohólico, así como las percepciones y creencias que rodean esta enfermedad. También, estamos interesados en aprender más sobre los patrones de comida y las perspectivas de una alimentación saludable. Las preguntas que le haremos a continuación son para explorar sus pensamientos y creencias en torno a la idea de las pruebas genéticas. Con el último conjunto de preguntas, estamos interesados en obtener sugerencias de miembros de la comunidad sobre cómo desarrollar programas para tratar la enfermedad del hígado graso no alcohólico y, específicamente, cómo podemos adaptar estos programas para satisfacer las necesidades de las mujeres de origen mexicano.

Su participación en esta entrevista es voluntaria y puede detenerse en cualquier momento que lo desee.

Cuando comencemos la entrevista, me gustaría grabar nuestra conversación. También estaré tomando algunas notas a medida que avancemos, pero la grabación de voz asegurará que no se me pase ningún detalle de nuestra conversación. ¿Tengo su permiso para grabar? Sus pensamientos y experiencias son realmente importantes para nosotros, por lo que queremos hacer nuestro mayor esfuerzo para capturar sus palabras con precisión. Me gustaría recordarle que todo lo que usted diga será confidencial. La grabación se almacenará de forma segura aquí en nuestra oficina (en la Universidad de Arizona –Centro de colaboración para la prevención y el tratamiento de enfermedades metabólicas). El equipo de investigación resumirá esta entrevista y utilizará su contribución para desarrollar resultados y conclusiones para la investigación, pero se eliminará toda información que pueda identificarla.

En este momento comenzare a grabar. [Comience a grabar]

Parte 1: Evaluación de la información /conocimiento, motivación y habilidades de comportamiento relacionadas con el riesgo para la enfermedad del hígado graso no alcohólico (EHGNA).

Conocimientos, percepciones, y creencias relacionadas con EHGNA y el riesgo para EHGNA

"Entonces, para esta primera sección, voy a hacerle algunas preguntas relacionadas con sus conocimientos y creencias en torno a la enfermedad del hígado graso no alcohólico (EHGNA)"

1. (Conocimiento /creencias) "¿Puede contarme un poco sobre lo que sabe acerca de la enfermedad del hígado graso no alcohólico (EHGNA)?"
    a. ¿Cómo se enteró de esto?
2. (Susceptibilidad percibida) ¿Cree que hay ciertas personas que tienen más probabilidad de obtener una enfermedad del hígado graso no alcohólico? O ¿Puede decirme qué podría hacer que alguien sea más propenso a "obtener" esta enfermedad?
3. ¿Hay algo que le gustaría compartir que aprendió de su participación en este estudio de investigación?
    a. ¿Aprendió algo diferente de lo que pensaba anteriormente?

Part 2: Experiencia recibiendo elastografía transitoria del hígado

"Gracias por compartir. Para esta próxima sección, me gustaría volver al día de su visita de estudio. Específicamente, quiero que piense en su experiencia al recibir el ultrasonido del hígado".

1. ¿Cuáles fueron algunos de sus pensamientos iniciales al ver los resultados de su ultrasonido hepático y hablar con el proveedor (médico)?
    a. ¿Fue útil la información que le dio el doctor?
    b. ¿Estría interesado en recibir otro ultrasonido gratis en el futuro?
2. ¿Compartió usted sus resultados, y si fue así, con quién?
3. ¿Tomó usted alguna acción específica como resultado de su ultrasonido o de su discusión con el proveedor (médico)?
4. Si hubiera tenido la oportunidad de aprender más sobre su riesgo de enfermedad del hígado graso no alcohólico extrayendo sangre o proporcionando una muestra de orina, ¿le habría interesado? ¿Por qué o por qué no?

Declaraciones finales

Supplementary File 3: Summary of Qualitative Themes, Codes, and Illustrative Quotes

Themes	Codes	Illustrative Quotes
Low levels of pre-study NAFLD awareness	"I didn't even know it existed"	*"I didn't know anything about it prior to getting the study done, this was when I started going online and reading more about it" (S, 50)*<br><br>*"To be honest with you, I didn't even know this disease existed"(S, 55)*<br><br>*"No, I didn't even know it existed" (S, 33)*<br><br>*I wasn't really aware of this topic until the day they were giving out backpacks, I learned information about this study there because I didn't know anything about it beforehand. I also knew that the liver sometimes fails, but I never really knew why." (S, 26)*<br><br>*"I actually wasn't aware of this disease. Before getting the study done, I didn't know about it." (S, 56)*
NAFLD awareness did not necessarily translate to greater NAFLD knowledge	Low pre-study knowledge related to NAFLD	*"I am going to be honest, beforehand, I had heard something about fatty liver, but I don't remember the specifics." (S, 61)*<br><br>*"I just know I've heard it mentioned" (E, 53)*<br><br>*"I know it existed but I never like see it on TV or anything like that like in a big way like 'oh my god you have to take care of it'" (E, 38)*
	"No one pays attention to the liver"	*"This is something one isn't aware of because one doesn't normally pay attention to the liver. Everyone says 'oh, I have diabetes because it runs in the family', but no one pays attention to the liver." (S, 26)*<br><br>*"You know like heart disease, diabetes and all that before your liver, there's not a lot of things known about it." (E, 38)*<br><br>*"And it was just like wow like I never would have thought…that, you know, I had the fatty liver or anything because like I said it's just kind of like forgotten about" (E, 50)*

	Medication use and fatty liver disease	*"To be honest with you, I didn't even know this disease existed. One of my brothers passed away three years ago from fatty liver disease due to a specific medication he was taking but I'm not quite sure. I'm not sure which types of medications are the ones that harmed his health. I have been paying a little bit more attention to this disease since that incident." (S, 55)*<br><br>*"It's not only due to drinking, taking certain medications also influence the development of this disease." (S, 39)*<br><br>*"And I think medication is a contribution to it too." (E, 42)*
Liver disease knowledge centered around cirrhosis	High levels of cirrhosis awareness	*Um…before this, I really didn't know anything about it…I had only heard of…like cirrhosis of the liver. That's it. I had never heard of fatty liver." (E, 50)*<br><br>*"Well, I didn't know this disease existed, specifically on this topic. I was under the impression that people who have liver problems are the ones who drink alcohol. This usually leads to cirrhosis, something of that sort. But I hadn't heard about fatty liver." (S, 43)*<br><br>*"But yeah, I don't know too much about where that disease goes, where it crosses, you know and to different you know cirrhosis, cancer, or anything like that." (E, 53)*<br><br>*"I was always under the impression that issues that had to do with the liver was due to drinking alcohol and I've also heard of liver cirrhosis and I thought it was because a person was drinking excessively." (S, 61)*
Family and media as predominant information sources related to liver disease	Family history	*"One of my brothers passed away three years ago from fatty liver disease due to a specific medication he was taking but I'm not quite sure. I'm not sure which types of medications are the ones that harmed his health. I have been paying a little bit more attention to this disease since that incident." (S, 55)*<br><br>*"Mostly from talking with you because I have no idea. My husband said he ate fatty liver disease but he didn't think it was a big thing. And then my daughter was diagnosed with it years ago and she's in her 20s." (E, 62)*

		"Well, um I mean…Personally I just recently um lost a family member to liver cancer and um I mean I know in the past he…I mean…let's just say he was a drunk. Drink like a lot. So I just heard that you know that affects it. So that's one of the…how I know a little bit like oh well maybe his drinking problem could have caused this cancer now, like developed it, or affected his liver." (E, 27)<br><br>*"A week ago, my husband's first cousin who was the only daughter passed away from a liver disease. But she had some other issues with her bones and joints, and I didn't really understand exactly what happened. I called my mother-in-law and she told me that the medications she was taking for her bones and joints affected her liver. Therefore, I don't remember what else happened, but I do know she passed away due to her liver." (S, 50)*
	Family history as motivation to learn more about liver disease	*"I've read it on my own searching just because my family has a history of liver disease. Both my parents passed away because of liver, my dad had liver cancer. And then my mother had liver cirrhosis…Because of their history and what went on with them which was recent, it made me want to look into it, so I started reading up on it." (E, 42)*<br><br>*"So my brother has it and I had never heard of it before that. And so I was like googling and I just…I just didn't really know too much other than just it's genetic, so." (E, 23)*<br><br>*"Um, just doing some reading online because I would always pop up on my…my um…e-mails sometimes. And my father died from liver disease. I didn't know him, but they say he wasn't an alcoholic. So that got me kind of thinking." (E, 56)*
	Media sources	*"I know it existed but I never like see it on TV or anything like that like in a big way like 'oh my god you have to take care of it.'" (E, 38)*<br><br>*"Um, just doing some reading online because I would always pop up on my…my um…e-mails sometimes. And my father died from liver disease. I didn't know him, but*

		they say he wasn't an alcoholic. So that got me kind of thinking." (E, 56)<br><br>*"I have heard about it [fatty liver] and I think I got it from watching movies on YouTube." (S, 37)*
	NAFLD education in school	*"And it's something that you don't learn about. Like I have a four-year degree and a lot of it was like I had to take nutrition classes. I had to take all these science classes. I had to take a lot of bio classes. Nobody ever told me this." (E, 26)*
Misperceptions related to risk for liver disease	Alcohol and risk of liver disease	*"I was under the impression that people who have liver problems are the ones who drink alcohol." (S, 43)*<br><br>*"I was always under the impression that issues that had to do with the liver was due to drinking alcohol and I've also heard of liver cirrhosis and I thought it was because a person was drinking excessively." (S, 61)*<br><br>*"I learned that you could have a fatty liver without alcohol. That's, I honestly like that was one of my biggest I guess breakthroughs." (E, 26)*
	Body size and fatty liver disease	*"I was really sure that I was going to have a fatty liver because I'm fat. So obviously you're going to have fat around your organs. But I think that's what it was." (E, 38)*<br><br>*"Yes, because now I know that people could get this disease as a result of not eating well rather than as a result of obesity and or due to alcohol. I didn't consider myself to be a big person especially not heavy to think that I could develop this disease." (S, 40)*<br><br>*"Yes. I have told them and even when I went to get the study done, I shared it with them, I shared with them the information I had learned here. And I'll repeat myself again, overweight people are not the only ones to develop this disease. We should take care of ourselves." (S, 50)*
	Effects of misperceptions on	*"Umm I did umm I was surprised considering that I don't drink and that I don't have any of those external…that would affect liver. I mean either drug use or whatever*

	perceived risk for liver disease	*that would affect. But I don't have those in my life." (E, 62)*<br><br>*"So I was thinking like well, I don't drink, I've never drank in my life. So it's like, well, it could also be a part of genetics or it could be like the way I eat. So, I know it wasn't triggering because of the alcohol because I don't consume it." (E, 27)*
